# The Effect of Photobiomodulation on Third Molar Wound Recovery: A Systematic Review with Meta-Analysis

**DOI:** 10.3390/jcm13185402

**Published:** 2024-09-12

**Authors:** Aldo Giansiracusa, Stefano Parrini, Nicola Baldini, Elena Bartali, Glauco Chisci

**Affiliations:** Oral Surgery Postgraduate School, Department of Medical Biotechnologies, University of Siena, 53100 Siena, Italy

**Keywords:** third molar, laser, photobiomodulation, wound healing, wound recovery, alveolar osteitis

## Abstract

**Objectives:** This study addresses the limited body of literature concerning the impact of photobiomodulation on complications following mandibular third molar extractions. **Methods:** A systematic literature review and meta-analysis of clinical studies that reported the use of photobiomodulation after mandibular third molar surgery was conducted based on PRISMA (Preferred Reporting Items for Systematic Reviews and Meta-Analyses) recommendations. The formulation of research questions followed the PICO model, and comprehensive strategies for record search and study selection were devised. The protocol was registered on PROSPERO (The Prospective Register of Systematic Reviews; no CRD42024511892). Two independent reviewers consulted four databases during the literature search: MEDLINE/PubMed, Google Scholar, Clinicaltrial.gov, and Cochrane Library databases without imposing any date restrictions. A search on the grey literature was carried out too (OpenGrey). Duplicate articles were eliminated. **Results:** After the initial screening, 18 studies were retained to be screened by the reviewers. The full texts of the identified studies were scrutinized for original data, and their related references were manually retrieved and checked for additional relevant studies. The available studies exhibit considerable heterogeneity, exploring various factors related to postoperative outcomes. Our meta-analysis primarily focuses on three key aspects: the incidence of alveolar osteitis (AO), mucosa repair/alveolar pocket healing, and bone repair. The resultant CI of the VAS scale was 98 to 99%. **Conclusions**: This meta-analysis underscores the need for further research in this domain, highlighting the existing heterogeneity among studies and the importance of a nuanced understanding of photobiomodulation’s multifaceted effects on postoperative complications.

## 1. Introduction

The extraction of the third molar, commonly known as the wisdom tooth, is one of the most common interventions in oral and maxillofacial surgery. This operation is often associated with a range of postoperative complications, including pain, swelling, limited mouth opening, and alveolar osteitis [1,2,3]. The most common third molar pathologies that lead to extraction are dysodontiasis and pericoronitis [4,5]; dysodontiasis refers to the inclusion characteristics of the affected tooth, while pericoronitis represents an infection of a unerupted impacted tooth that may arise after dysodontiasis [5,6].

With regards to the postoperative outcomes and clinical symptoms after third molar extraction, much effort has been exerted by researchers to find strategies to reduce postoperative swelling and pain [7,8,9,10]. With regards to the possibility of the development of postoperative infection and alveolar osteitis, the role of antibiotics has been discussed too, with many controversial aspects [11,12,13]. Photobiomodulation—also named Low-Level Laser Therapy—is a therapy based on the use of low-power light radiation that positively influences biological processes and the healing of tissues. This therapy is based on a photochemical mechanism whereby energy is transferred to intracellular mitochondrial chromophores, i.e., molecules that are capable of light absorption such as endogenous porphyrins and some components of the respiratory chain like cytochrome-C oxidase. These molecules can transfer the absorbed laser energy to mitochondria, where it is converted into metabolic energy through the respiratory chain, producing adenosine triphosphate (ATP). The light absorption by respiratory chain components causes a short-term activation of the respiratory chain itself and the oxidation of nicotinamide adenine dinucleotide (NADH), leading to changes in both mitochondrial and cytoplasmic redox states. It is helpful in reducing postoperative symptoms because it induces different biological changes. Recently, Souza et al. [14] reported good results with photobiomodulation after tooth extraction, suggesting an increased recovery compared to their control group; their suggestion was to avoid antibiotics administration. In the ongoing effort to enhance the postoperative experience for patients undergoing this procedure, photobiomodulation has garnered attention as a potential therapeutic strategy. Photobiomodulation, based on the application of low-power light radiation, has demonstrated positive effects in various medical contexts, including the reduction of inflammation and the promotion of tissue healing. Despite some preliminary evidence suggesting benefits from the use of photobiomodulation in the context of third molar extraction, the scientific literature is still characterized by a variety of results and methodological approaches, such as intraoral or extraoral application, time, and device modality [15].

Therefore, due to the lack in the current literature related to some possible benefits of photobiomodulation after mandibular third molar surgery outcomes, such as bone repair, mucosal repair, and the influence of incidence of alveolar osteitis, it is essential to conduct a systematic review and meta-analysis to synthesize existing evidence, assess the table quality of studies, and provide an overall evaluation of these aspects. This review aims to consolidate current knowledge by identifying emerging trends and providing a solid scientific foundation for the implementation of photobiomodulation as an integral part of postoperative clinical practices. Through a thorough assessment of available research, we aim to contribute to the understanding of the potential benefits of photobiomodulation in optimizing the clinical recovery of the alveolar pocket for patients undergoing this common dental surgical procedure. Our hypothesis is that photobiomodulation could influence and enhance the bone and mucosal repair and reduce the incidence of alveolar osteitis after mandibular third molar surgery. This aspect could positively influence the postoperative recovery and reduce symptoms. For this reason, we performed a systematic review with meta-analysis, registered a priori on PROSPERO (The Prospective Register of Systematic Reviews; no CRD42024511892).

## 2. Materials and Methods

The research protocol adhered to the guidelines set forth in the Preferred Reporting Items for Systematic Reviews and Meta-Analyses (PRISMA) statement [16]. The formulation of research questions followed the PICO model, and comprehensive strategies for record search and study selection were devised. The protocol was registered on PROSPERO (The Prospective Register of Systematic Reviews; no CRD42024511892). Review methods were established prior to conducting the review.

### 2.1. Eligibility Criteria

All studies investigating the reduction of postoperative complications following third molar extraction with the use of photobiomodulation were included in the present protocol.

Inclusion criteria were defined according to the PICO(S) method:

- Population: individuals with a need for third molar extraction;
- Intervention: the extraction of the third molar(s) and the use of photobiomodulation;
- Comparison: the recovery of the alveolar pocket after third molar extraction;
- Outcome: the primary outcome of the protocol was defined as the alveolar pocket healing 7 days after the third molar extraction with photobiomodulation. The secondary outcome of the protocol was defined as the incidence of postoperative alveolar osteitis with photobiomodulation;
- Studies: randomized controlled clinical trials in the English or Italian language.

The exclusion criteria were defined as follows: (1) there was no extraction of mandibular third molars; (2) photobiomodulation was not used during the surgery; (3) no data about alveolar bone pocket healing; (4) studies not conducted in English nor in Italian.

### 2.2. Information Sources

**Figure 1:**
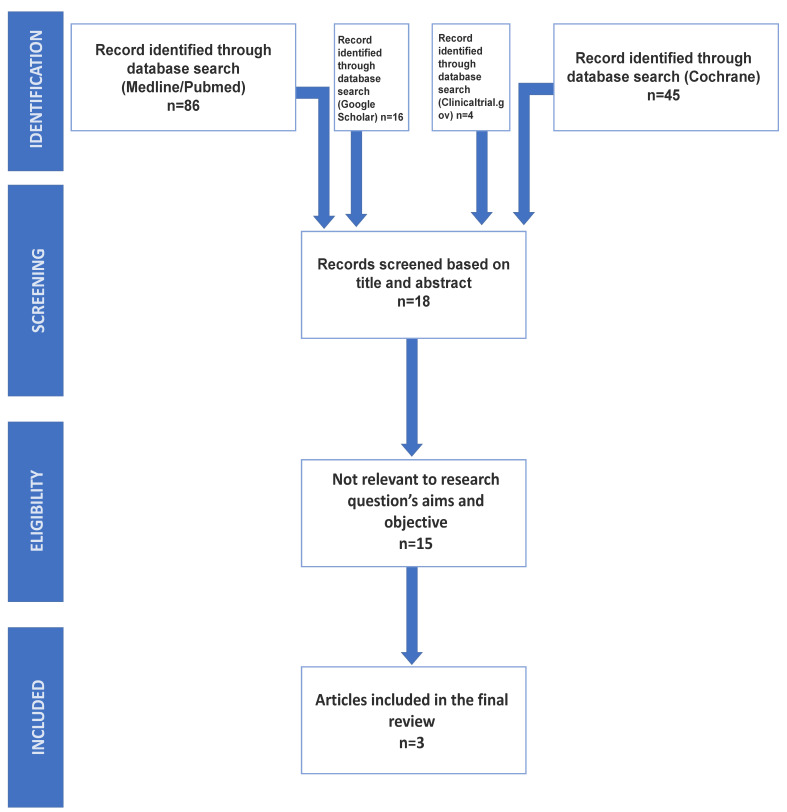
The presented PRISMA flow diagram indicates the steps that were followed to include n = 3 eligible studies for the meta-analysis.

The search strategy included the screening of electronic databases. The screening and inclusion stages were reported following the PRISMA flow diagram [1] (Figure 1).

### 2.3. Research Strategy

Electronic searches for relevant articles were conducted on the MEDLINE/PubMed, Google Scholar, Clinicaltrial.gov, and Cochrane Library databases without imposing any date restrictions. The search utilized the keywords (Third molar) or (wisdom tooth) AND (Photobiomodulation) or (laser) from 2015 up to 31 January 2024. A search on the grey literature was carried out too (OpenGrey). No filters were applied to limit the scope of the research.

### 2.4. Study Selection

These articles underwent an initial screening process, involving the examination of titles and abstracts while eliminating any duplications. The complete texts of the potentially eligible studies, based on their title and abstract, were obtained and meticulously reviewed by the same set of reviewers to verify that they met the main objectives of this study. The full texts of the identified studies were scrutinized for original data, and their related references were manually retrieved and checked for additional relevant studies. Inclusion criteria were strictly applied, and, after the second screening, the selected studies were considered eligible and directly relevant to the research questions of the study.

### 2.5. Data Collection Process

Prior to the selection of the articles, the eligibility criteria were discussed and the selection was performed. In cases of a divergence of opinion, a third expert reviewer was consulted to arrive at a final decision regarding inclusion or exclusion. The characteristics of the included studies encompassed participant age and gender, follow-up, alveolar osteitis, mucosal repair/alveolar pocket healing, and Visual Analog Scale (VAS).

### 2.6. Data Items

The primary outcome of the protocol was defined as the alveolar pocket healing 7 days after the third molar extraction with photobiomodulation. The secondary outcome of the protocol was defined as the incidence of postoperative alveolar osteitis with photobiomodulation.

### 2.7. Quality Assessment

The overall quality of the included studies was assessed through the Newcastle Ottawa Scale (NOS) for cohort studies [17]. It includes three categories (selection, comparability, and outcome), with an overall score ranging between 0 (low quality) and 9 (high quality) points. Studies with an NOS score greater than 6 were deemed to be of moderate to high quality (Table 1).

The risk of bias in randomized controlled trials was evaluated using the revised Cochrane Risk of Bias tool (RoB2) [18] (Table 2).

Each study was evaluated through five domains:

1. Randomization process;
2. Deviation from intended intervention;
3. Missing outcome data;
4. Measurement of the outcome;
5. Selection of reported results.

The assessment of the risk of bias resulted in three categories:

- High risk: whenever at least one domain was defined as “high risk of bias”;
- Some concerns: whenever at least one domain raised “some concerns”;
- Low risk: whenever no domain was defined as being “low risk of bias” or raising “some concerns”.

The Cochran Q statistic [19] and the I^2^ index were used in order to estimate heterogeneity across studies. Between-study variance was estimated using the T2 parameter. A meta-analysis of the included studies was conducted through an inverse variance analysis using the DerSimonian and Laird random effects model [20,21].

Moreover, publication bias was assessed through the Egger test [22]. Values of *p* < 0.05 were considered statistically significant.

#### 2.7.1. Summary Measures

Data were pooled for both qualitative and quantitative analysis. The only evaluation test that was common to all three included studies was the VAS scale. The analysis was performed using the results of the VAS scale at day 7 in the test group from each study. The CI was calculated for these values.

#### 2.7.2. Synthesis of Results and Additional Analyses

Statistical analysis was carried out through https://www.stata.com [23].

## 3. Results

### 3.1. Study Selection

Following the conclusion of the research conducted on the two electronic databases, a total of 151 articles were identified: 86 from MEDLINE/PubMed, 45 from Cochrane Library, 16 from Google Scholar, and 4 from Clinicaltrial.gov. These articles underwent an initial screening process, involving the examination of titles and abstracts while eliminating any duplications. After this initial screening, 18 studies were retained to be screened by the reviewers [24,25,26,27,28,29,30,31,32,33,34,35,36,37,38,39,40,41]. The full texts of the identified studies were scrutinized for original data, and their related references were manually retrieved and checked for additional relevant studies. Inclusion criteria were strictly applied, and, after the second screening, the selected studies were considered eligible and directly relevant to the research questions of the study.

The complete texts of these potentially eligible studies, based on their title and abstract, were obtained and meticulously reviewed by two reviewers to verify that they met the main objectives of this study.

The full texts of the identified studies were scrutinized for original data, and their related references were manually retrieved and checked for additional relevant studies. Inclusion criteria were strictly applied, and, after the second screening, only 3 out of the 18 studies were considered eligible and directly relevant to the research questions of the study.

### 3.2. Newcastle Ottawa

All the included studies were found to be of moderate to high quality (Table 1). Only four of the evaluating parameters of NOS were always positive between the three studies during the manual coding: ‘Case Definition’, ‘Representativnes of the Cases’, ‘The assestment of outcome’ and ‘Adequacy of Followup of Cohorts’.

Two studies, those by Gururaj et al. [25] and Nejat et al. [24], obtained a total score of 6 on the NOS scale, indicating a moderate level of methodological quality. Pereira et al. [26] achieved a total score of 7, indicating a slightly higher level of quality compared to the other two studies (Table 1).

**Table 1:** The table presents the assessment of methodological quality in the included studies using the Newcastle Ottawa Scale (NOS). The scale evaluates three main categories: the selection of studies, comparability, and outcome assessment. Total scores range from 0 to 9, with higher scores indicating higher methodological quality [18].

Author (Year)	Selection	Comparability	Outcome	Total Score
Gururaj et al. (2022) [25]	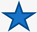 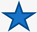 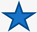	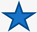	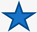 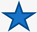	6
Nejat et al. (2021) [24]	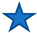 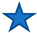	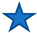 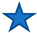	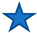 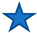	6
Pereria et al. (2022) [26]	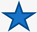 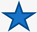 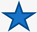	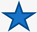	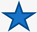 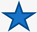 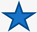	7

### 3.3. Overall Bias

**Figure 2:**
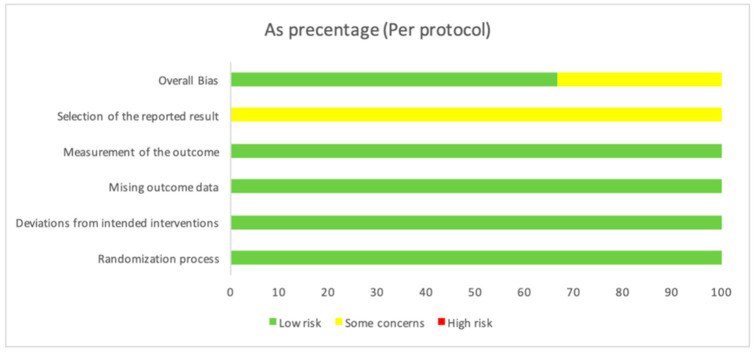
The overall bias as a percentage. In the first row, there is a 66.7% value in green, indicating a low risk, while, in yellow, there is a value of 33.3%, indicating some concerns. The second row shows a value of 100% in yellow, indicating some concerns about the “Selection of the reported result”. For the other evaluated variables, there is a value of 100, indicating a low risk.

The three analyzed studies (Gururaj et al. [25], Nejat et al. [24], and Pereira et al. [26]) investigate the effects of photobiomodulation therapy on third molar extraction procedures. Overall, all three studies exhibit a low risk of bias, indicating adequate randomization, the effective control of deviations from planned interventions, minimal missing data, and accurate outcome measurement (Table 2). However, there are some concerns regarding the selection of reported outcomes in each study. Despite these potential limitations, the overall bias assessment for each study is favorable (Table 2), suggesting that the obtained results are reliable in the context of evaluating the efficacy of photobiomodulation therapy in third molar extraction procedures (Figure 2).

**Table 2:** Y = yes; N = no; PY = probably yes; PN = probably no; NA = not applicable; NI = no information.

*Unique ID*	*1*	*Study ID*	*Gururaj* et al., *2022* [25]	
***Aim***	adhering to intervention (the ‘per-protocol’ effect)	**The effect of adhering to intervention…**	failures in implementing the intervention that could have affected the outcome	
***Source***	Journal article(s)			
***Outcome***	asses the effect of preoperative as well as postoperative photobiomodulation on healing as well pain at mandibular third molar extraction sockets	**Weight**	1	
***Domain***	**Signalling question**			**Response**
***Bias arising from the randomization process***	1.1 Was the allocation sequence random?			Y
	1.2 Was the allocation sequence concealed until participants were enrolled and assigned to interventions?			PY
	1.3 Did baseline differences between intervention groups suggest a problem with the randomization process?			PN
	**Risk of bias judgement**			**Low**
***Bias due to deviations from intended interventions***	2.1 Were participants aware of their assigned intervention during the trial?			N
	2.2 Were carers and people delivering the interventions aware of participants’ assigned intervention during the trial?			PN
	2.3. [If applicable:] If Y/PY/NI to 2.1 or 2.2: Were important non-protocol interventions balanced across intervention groups?			NA
	2.4. [If applicable:] Were there failures in implementing the intervention that could have affected the outcome?			
	2.5. [If applicable:] Was there non-adherence to the assigned intervention regimen that could have affected participants’ outcomes?			NA
	2.6. If N/PN/NI to 2.3, or Y/PY/NI to 2.4 or 2.5: Was an appropriate analysis used to estimate the effect of adhering to the intervention?			NA
	**Risk of bias judgement**			**Low**
***Bias due to missing outcome data***	3.1 Were data for this outcome available for all, or nearly all, participants randomized?			Y
	3.2 If N/PN/NI to 3.1: Is there evidence that result was not biased by missing outcome data?			NA
	3.3 If N/PN to 3.2: Could missingness in the outcome depend on its true value?			NA
	3.4 If Y/PY/NI to 3.3: Is it likely that missingness in the outcome depended on its true value?			NA
	**Risk of bias judgement**			**Low**
***Bias in measurement of the outcome***	4.1 Was the method of measuring the outcome inappropriate?			PN
	4.2 Could measurement or ascertainment of the outcome have differed between intervention groups?			PN
	4.3 Were outcome assessors aware of the intervention received by study participants?			PN
	4.4 If Y/PY/NI to 4.3: Could assessment of the outcome have been influenced by knowledge of intervention received?			NA
	4.5 If Y/PY/NI to 4.4: Is it likely that assessment of the outcome was influenced by knowledge of intervention received?			NA
	**Risk of bias judgement**			**Low**
***Bias in selection of the reported result***	5.1 Were the data that produced this result analysed in accordance with a pre-specified analysis plan that was finalized before unblinded outcome data were available for analysis?			PY
	5.2 … multiple eligible outcome measurements (e.g., scales, definitions, time points) within the outcome domain?			Y
	5.3 … multiple eligible analyses of the data?			NI
	**Risk of bias judgement**			**Some concerns**
***Overall bias***	**Risk of bias judgement**			**Some concerns**
***Unique ID***	2	**Study ID**	Nejat et al., 2021 [24]	
***Aim***	adhering to intervention (the ‘per-protocol’ effect)	**The effect of adhering to intervention…**	failures in implementing the intervention that could have affected the outcome	
***Source***	Company-owned trial registry record (e.g., GSK Clinical Study Register record)			
***Outcome***	effectivness of photobiomodulation therapy for the prevention of incidence of Alveolar osteitis and post-operative pain following third molar surgery	**Weight**	1	
***Domain***	**Signalling question**			**Response**
***Bias arising from the randomization process***	1.1 Was the allocation sequence random?			Y
	1.2 Was the allocation sequence concealed until participants were enrolled and assigned to interventions?			Y
	1.3 Did baseline differences between intervention groups suggest a problem with the randomization process?			PN
	**Risk of bias judgement**			**Low**
***Bias due to deviations from intended interventions***	2.1 Were participants aware of their assigned intervention during the trial?			N
	2.2 Were carers and people delivering the interventions aware of participants’ assigned intervention during the trial?			N
	2.3. [If applicable:] If Y/PY/NI to 2.1 or 2.2: Were important non-protocol interventions balanced across intervention groups?			NA
	2.4. [If applicable:] Were there failures in implementing the intervention that could have affected the outcome?			PN
	2.5. [If applicable:] Was there non-adherence to the assigned intervention regimen that could have affected participants’ outcomes?			NA
	2.6. If N/PN/NI to 2.3, or Y/PY/NI to 2.4 or 2.5: Was an appropriate analysis used to estimate the effect of adhering to the intervention?			NA
	**Risk of bias judgement**			**Low**
***Bias due to missing outcome data***	3.1 Were data for this outcome available for all, or nearly all, participants randomized?			Y
	3.2 If N/PN/NI to 3.1: Is there evidence that result was not biased by missing outcome data?			NA
	3.3 If N/PN to 3.2: Could missingness in the outcome depend on its true value?			NA
	3.4 If Y/PY/NI to 3.3: Is it likely that missingness in the outcome depended on its true value?			NA
	**Risk of bias judgement**			**Low**
***Bias in measurement of the outcome***	4.1 Was the method of measuring the outcome inappropriate?			PN
	4.2 Could measurement or ascertainment of the outcome have differed between intervention groups?			N
	4.3 Were outcome assessors aware of the intervention received by study participants?			N
	4.4 If Y/PY/NI to 4.3: Could assessment of the outcome have been influenced by knowledge of intervention received?			NA
	4.5 If Y/PY/NI to 4.4: Is it likely that assessment of the outcome was influenced by knowledge of intervention received?			NA
	**Risk of bias judgement**			**Low**
***Bias in selection of the reported result***	5.1 Were the data that produced this result analysed in accordance with a pre-specified analysis plan that was finalized before unblinded outcome data were available for analysis?			PN
	5.2 … multiple eligible outcome measurements (e.g., scales, definitions, time points) within the outcome domain?			Y
	5.3 … multiple eligible analyses of the data?			NI
	**Risk of bias judgement**			**Some concerns**
***Overall bias***	**Risk of bias judgement**			**Low**
***Unique ID***	3	**Study ID**	Pereira 2022 [26]	
***Aim***	adhering to intervention (the ‘per-protocol’ effect)	**The effect of adhering to intervention…**	failures in implementing the intervention that could have affected the outcome	
***Source***	Journal article(s)			
***Outcome***	evaluate photobiomodulation therapy with the association of red and infra-red laser therapy in the healing of the post-extraction sockets of third lower molar	**Weight**	1	
***Domain***	**Signalling question**			**Response**
***Bias arising from the randomization process***	1.1 Was the allocation sequence random?			Y
	1.2 Was the allocation sequence concealed until participants were enrolled and assigned to interventions?			PY
	1.3 Did baseline differences between intervention groups suggest a problem with the randomization process?			PN
	**Risk of bias judgement**			**Low**
***Bias due to deviations from intended interventions***	2.1 Were participants aware of their assigned intervention during the trial?			N
	2.2 Were carers and people delivering the interventions aware of participants’ assigned intervention during the trial?			N
	2.3. [If applicable:] If Y/PY/NI to 2.1 or 2.2: Were important non-protocol interventions balanced across intervention groups?			NA
	2.4. [If applicable:] Were there failures in implementing the intervention that could have affected the outcome?			N
	2.5. [If applicable:] Was there non-adherence to the assigned intervention regimen that could have affected participants’ outcomes?			NA
	2.6. If N/PN/NI to 2.3, or Y/PY/NI to 2.4 or 2.5: Was an appropriate analysis used to estimate the effect of adhering to the intervention?			NA
	**Risk of bias judgement**			**Low**
***Bias due to missing outcome data***	3.1 Were data for this outcome available for all, or nearly all, participants randomized?			Y
	3.2 If N/PN/NI to 3.1: Is there evidence that result was not biased by missing outcome data?			NA
	3.3 If N/PN to 3.2: Could missingness in the outcome depend on its true value?			NA
	3.4 If Y/PY/NI to 3.3: Is it likely that missingness in the outcome depended on its true value?			NA
	**Risk of bias judgement**			**Low**
***Bias in measurement of the outcome***	4.1 Was the method of measuring the outcome inappropriate?			PN
	4.2 Could measurement or ascertainment of the outcome have differed between intervention groups?			PN
	4.3 Were outcome assessors aware of the intervention received by study participants?			PN
	4.4 If Y/PY/NI to 4.3: Could assessment of the outcome have been influenced by knowledge of intervention received?			NA
	4.5 If Y/PY/NI to 4.4: Is it likely that assessment of the outcome was influenced by knowledge of intervention received?			NA
	**Risk of bias judgement**			**Low**
***Bias in selection of the reported result***	5.1 Were the data that produced this result analysed in accordance with a pre-specified analysis plan that was finalized before unblinded outcome data were available for analysis?			PY
	5.2 … multiple eligible outcome measurements (e.g., scales, definitions, time points) within the outcome domain?			PY
	5.3 … multiple eligible analyses of the data?			NI
	**Risk of bias judgement**			**Some concerns**
***Overall bias***	**Risk of bias judgement**			**Low**

### 3.4. Cochran Q Statistic & I^2^ Index

After assigning numerical values to the responses (“YES” = 1 and “NO” = 0) for each study (Table 2), the Q test is computed, yielding a result of 0. This indicates the absence of significant heterogeneity among the studies. However, it is important to note that, with so few studies and without additional information on variance, the ability to detect heterogeneity may be limited. Subsequently, an attempt was made to calculate the I^2^ index to quantify heterogeneity, but, due to the Q-test being equal to zero, the standard formula is not applicable. Therefore, it is commonly assumed that I^2^ is 0%, indicating the absence of heterogeneity. In summary, based on the test results, it appears that there is no significant heterogeneity among the studies included in the meta-analysis.

Based on the data provided by the three studies (Gururaj et al. [25], Nejat et al. [24], and Pereira et al. [26]), there seems to be a unanimous consensus on the enhanced efficacy of photobiomodulation in clinical outcomes after mandibular third molar extraction (Table 2). All three studies report an affirmative response, indicated by “I” (yes) to the question of whether photobiomodulation is effective in this context.

### 3.5. Risk of Bias

All three studies were generally assessed with a low risk of bias, with minor concerns in specific aspects, and seemed to offer valid and reliable contributions to the examined topic (Table 3). The research by Gururaj et al. [25] examined the effects of preoperative and postoperative photobiomodulation on healing and pain in mandibular third molar extraction sites. Despite some concerns about deviations from the intended intervention and missing data, the adherence to the intervention’s effect is rated as low risk. Overall bias is assessed as low, suggesting the study’s reliability in evaluating the photobiomodulation effect. The investigation by Nejat et al. [24] focused on the effectiveness of photobiomodulation therapy in preventing alveolar osteitis and postoperative pain after third molar extraction. Despite some concerns regarding the selection of reported results, the adherence to the intervention’s effect is assessed as low risk. Most bias assessments are judged as low, reinforcing the overall validity of the study. The investigation by Nejat et al. [24] focused on the effectiveness of photobiomodulation therapy in preventing alveolar osteitis and postoperative pain after third molar extraction. Despite some concerns regarding the selection of reported results, the adherence to the intervention’s effect is assessed as low risk. Most bias assessments are judged as low, reinforcing the overall validity of the study.

### 3.6. Quantitative Analysis

Regarding the effects of photobiomodulation on alveolar osteitis, only the study by Nejat et al. [24] focused on the extraction of the mandibular third molar and the onset of alveolar osteitis (AO). This study revealed that, out of 160 performed extractions, 35 cases exhibited AO, with an incidence rate of 21.87%. The PBM therapy group recorded an AO incidence rate of 0.52% compared to the control group treated with simulated PBM therapy.

Concerning the effects on mucosa repair/alveolar pocket healing, only two studies reported results: Gururaj et al. [25] and Pereira et al. [26]. In the study by Gururaj et al. [25], the authors demonstrated that, on the 7th day and on the 21st day, the healing index was higher in the test group than the control group, while, in the study by Pereira et al. [26], they found that photobiomodulation therapy reduced the edema and improved the repair of the oral mucosa.

The effect of photobiomodulation on bone repair was only studied by Pereira et al. [26]. They found that the tomographic evaluation showed progressive improvement in bone repair in both groups at 90 days compared to 7 days; however, there were no differences between the groups regarding repair pattern, bone density, and fractal dimension in relation to the post-extraction sockets treated with photobiomodulation therapy and the control alveoli.

The only homogeneous variable among the three included studies for which it was possible to calculate the CI was the VAS scale. The resultant CI of the VAS scale was 98 to 99%.

### 3.7. Prospective Studies

The three included studies are all prospective studies that provide valuable insights into the effects of photobiomodulation (PBM) therapy in different clinical contexts. In the study by Gururaj et al. [25], the focus was on general healing outcomes. The experimental group, subjected to PBM therapy, exhibited a significant improvement in both the healing index and Visual Analog Scale (VAS) scores compared to the control group on the 7th and 21st days. Nejat et al. [24] concentrated on patients undergoing dental extractions, particularly exploring the correlation with alveolar osteitis (AO). The PBM group demonstrated a significantly lower incidence of AO compared to the sham PBM group, indicating a potential preventive role. Additionally, both groups experienced a noteworthy reduction in pain intensity over time, with distinct differences in VAS scores observed on days 2–5 postoperation. The practical implication was a reduced need for analgesics in the PBM therapy group, highlighting its clinical relevance. The research by Pereira et al. [26] delved into the specific application of dual-wavelength PBM therapy in post-extraction alveolar repair. The results showcased positive effects, such as reduced edema and enhanced mucosal healing at short intervals. While there were no significant impacts on pain or bleeding, the long-term evaluation at 90 days demonstrated progressive bone repair in both the treated and control groups. The absence of distinctions in repair patterns, bone density, or fractal dimensions emphasized the overall similarity in outcomes. Collectively, these studies contribute to the argument that PBM therapy holds promise in diverse clinical scenarios, showing positive outcomes in general healing, the prevention of complications like AO, and specific applications in post-extraction alveolar repair. The findings underscore the potential benefits of incorporating PBM into clinical practices for enhanced patient outcomes.

Summarizing the characteristics of these 3 prospective studies (Table 4), we observe that the total number of participants in the 3 included studies was 124, comprising both males and females, all aged 18 years or older. The follow-up period across the three studies ranged from a minimum of 7 days to a maximum of 90 days. While all three studies appear to show some positive effects on post-extraction dental healing in using photobiomodulation therapy, there are differences in the measured variables and reported outcomes. Gururaj et al. [25] and Nejat et al. [24] highlighted effectiveness in reducing alveolar osteitis and improvements based on the Visual Analog Scale (VAS), whereas Pereira et al. [26] focused on mucosal healing and long-term bone repair.

## 4. Discussion

Postoperative complications after mandibular third molar surgery represent a great matter of interest, both for researchers and for clinicians. The recent innovation of the use of photobiomodulation represents a useful instrument in the hands of oral surgeons in order to reduce postoperative morbidity. On the basis of our systematic review, as deduced from both qualitative and quantitative analyses, the effect of photobiomodulation therapy on alveolar healing following the extraction of lower third molars remains a topic requiring further studies to establish its efficacy. The only consistently homogeneous data from the three studies included in the review was the Visual Analog Scale (VAS), which leans in favor of photobiomodulation therapy after mandibular third molar surgery.

The role of photobiomodulation after third molar extraction was previously reported by Lacedras-Santos [42] and Camolesi et al. [43], who examined its correlation with photobiomodulation. In both studies, a reduction in pain was observed as a result of photobiomodulation application too. The results of this study in terms of VAS are in line with these previous articles.

The innovative aspects of the present systematic review compared to previous systematic reviews were the research into the possible influence of photobiomodulation on mucosal repair, bone repair, and the reduction of alveolar osteitis after mandibular third molar surgery [42,43].

With regards to these innovative parameters, Gururaj et al. [25] demonstrated that the healing index was higher in the test group than control group, while Pereira et al. [26] found that photobiomodulation therapy reduced the edema and improved the repair of the oral mucosa.

The effect of photobiomodulation on bone repair was only studied by Pereira et al. [26]. They found that the tomographic evaluation showed progressive improvement in bone repair; however, there were no differences between the groups regarding repair pattern, bone density, and fractal dimension in relation to the post-extraction sockets treated with photobiomodulation therapy versus the control sites.

The effect of photobiomodulation and the influence on alveolar osteitis was only reported by Nejat et al. [24]; in this research paper, the authors reported a significant difference in the photobiomodulation group compared to the control group, with a lower alveolar osteitis rate in the photobiomodulation group. This aspect is very interesting and was one of the reasons that motivated the present research.

Souza et al. [14] reported good results in terms of antimicrobial control with photodynamic therapy after molar extraction: the suggestion that photobiomodulation could reduce the use of antibiotics in third molar surgery is a great matter of interest for ecological antibiotics administration and sustainability [44,45,46].

With regards to the Pergolini et al., 2022 [47] paper, this paper lacks any investigation regarding the three outcome variables of alveolar osteitis, mucosa healing, and bone repair. Further, Pergolini et al. is a simple but interesting narrative review, but without a current quality evaluation of the studies or a meta-analysis. Further, Pergolini et al. used different search databases and lower-quality statistical tests [47].

However, the present review was unable to retrieve sufficient scientific evidence with regards to complications such as alveolar osteitis or bone repair, as there is still limited scientific evidence supporting the effectiveness of photobiomodulation after mandibular third molar surgery.

The strengths of the present research are found in the strict paper selection protocol, the PROSPERO validation, and the accurate statistical analysis. Further, the topic of this study—third molar surgery research—is very selective but of paramount importance, as postoperative alveolar osteitis represents a great matter of interest. The main limitations of the present research are the reduced number of keywords for research and the limited number of studies at the end of the PRISMA selection that evaluated bone and mucosal repair and alveolar osteitis after photobiomodulation after third molar surgery; however, this element underlines a lack in the international literature.

Future research should emphasize (with randomized clinical trials) the study of alveolar osteitis incidence after mandibular third molar surgery and the role of photobiomodulation.

## 5. Conclusions

On the basis of the present study, we confirm that photobiomodulation is a reliable method to reduce VAS values after mandibular third molar surgery. Unfortunately, there is limited literature available on the effects of photobiomodulation on alveolar osteitis, bone repair, and mucosal repair following the extraction of mandibular third molars. Moreover, the existing studies are highly heterogeneous, as they use different postoperative values.

Accurate case selection and operative protocol for future studies are of paramount importance to reduce heterogeneity.

## Figures and Tables

**Table 3:** D1 = Randomization process; D2 = Deviations from the intended interventions; D3 = Missing outcome data; D4 = Measurement of the outcome; D5 = Selection of the reported result.

Per-Protocol	Unique ID	Study ID	Experimental	Comparator	Outcome	Weight	D1	D2	D3	D4	D5	Overall
	1	Gururaj et al., 2022 [25]	NA	NA	asses the effect of preoperative as well as postoperative photobiomodulation on healing as well pain at mandibular third molar extraction sockets	1	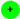	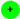	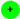	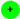	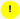	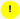
	2	Nejat et al., 2021 [24]	NA	NA	effectivness of photobiomodulation therapy for the prevention of incidence of Alveolar osteitis and post-operative pain following third molar surgery	1	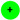	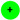	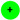	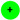	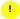	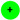
	3	Pereira 2022 [26]	NA	NA	evaluate photobiomodulation therapy with the association of red and infra-red laser therapy in the healing of the post-extraction sockets of third lower molar	1	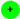	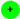	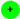	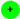	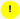	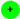

**Table 4:** The main characteristics of the included prospective studies.

Author (Year)	Mean Age (Range), y	Follow-Up	Partecipants (M/F), n	Effect on Reducing Alveolar Osteitis	Effect on Mucosal Repair/Alveolar Pocket Healing	Improvement Based Visual Analogue Score (VAS)	Effect on Bone Repair
Gururaj et al. (2022) [25]	NR	21-days	26 (14 + 12)	NR	YES	YES	NR
Nejat et al. (2021) [24]	24+/− 4.08	7-days	80 (29 + 51)	YES	NR	YES	NR
Pereira et al. (2022) [26]	>18	90-days	18	NR	YES	NO	YES

NR = no response.
